# Expression of a hyperthermophilic endoglucanase in hybrid poplar modifies the plant cell wall and enhances digestibility

**DOI:** 10.1186/s13068-018-1224-7

**Published:** 2018-08-16

**Authors:** Yao Xiao, Xuejun He, Yemaiza Ojeda-Lassalle, Charleson Poovaiah, Heather D. Coleman

**Affiliations:** 1 Biology Department, Syracuse University, Syracuse, NY 13244 USA; 2 Present Address: Scion, Te Papa Tipu Innovation Park, 49 Sala Street, Rotorua, 3010 New Zealand

**Keywords:** Hyperthermophilic cellulase, Transgenic poplar, Cell wall composition, Saccharification, Biofuel production

## Abstract

**Background:**

Expression of glycosyl hydrolases in lignocellulosic biomass has been proposed as an alternative to improve efficiency of cellulosic ethanol production. *In planta* production of hyperthermophilic hydrolytic enzymes could prevent the detrimental effects often seen resulting from the expression of recombinant mesophilic enzymes to plant hosts. Utilizing lignocellulosic feedstocks to produce hyperthermophilic hydrolases provides additional benefits for ethanol production in the way of transgenic feedstocks serving as both enzyme providers and cellulosic substrates.

**Results:**

In this study, transgenic hybrid poplar (*Populus alba *× *grandidentata*) was generated to express a hyperthermophilic endoglucanase from *Thermotoga neapolitana* with an optimal temperature over 100 °C. Functional hyperthermoactive endoglucanase was successfully produced in the transgenic events, and altered phenotypic growth was observed in transgenic lines. Moreover, the line with the highest *Tn*CelB expression in both leaf and developing xylem had reduced lignin content and cellulose crystallinity, resulting in a more digestible cell wall. The activation of *Tn*CelB by a post-harvest heat treatment resulted in enhanced saccharification efficiencies of transgenic poplar lines with moderate *Tn*CelB expression and without alteration of cellulose and lignin when not heat-treated. *In planta* high-level overexpression of a hyperthermophilic endoglucanase paired with heat treatment following harvest, resulted in biomass that was comparable with wild-type lines that underwent a traditional pretreatment for saccharification.

**Conclusions:**

Overexpression of hyperthermophilic endoglucanase in feedstock had impacts on plant growth and cell wall composition, especially when the enzyme was highly expressed. Improved glucan saccharification efficiencies from transgenic lines before and after heat treatment could reduce both the economic and environmental costs associated with ethanol production from lignocellulosic biomass.

**Electronic supplementary material:**

The online version of this article (10.1186/s13068-018-1224-7) contains supplementary material, which is available to authorized users.

## Background

The drive to replace fossil fuels with sustainable energy sources has resulted in increased demand for ethanol to supplement gasoline since the late 1970s [1]. While the production of grain-derived ethanol has been criticized as a potential threat to food supply and for its debatable social benefits [2], the utilization of lignocellulosic biomass, one of the most abundant energy resources on the planet, provides an alternative for ethanol production. The secondary cell wall of lignocellulosic biomass contains abundant polysaccharides such as cellulose and hemicellulose that can be hydrolyzed into monosaccharides for fermentation into ethanol [3]. Currently, low efficiency and high conversion costs prevent large-scale production of lignocellulosic ethanol.

Degradation of the plant cell wall requires thermochemical pretreatment followed by enzymatic hydrolysis. Cellulose bundles are embedded within a complex matrix of hemicellulose and lignin, and pretreatment is necessary to allow access to the carbohydrates of the cell wall. Pretreatment utilizes high temperatures, corrosive chemicals, or ionic liquids to break the bonds among the three components, thereby increasing accessibility of hydrolytic enzymes to cellulose [4]. These glycosyl hydrolases, which include a suite of enzymes that breakdown cellulose and hemicellulose, are commonly produced in microorganisms. Pretreatments requiring harsh thermochemical conditions paired with the high cost of microbe-derived enzymes continue to make lignocellulosic ethanol financially non-competitive compared with corn ethanol.

*In planta* expression of cellulolytic enzymes is an alternative to producing the enzymes in microorganisms. While proof of concept has been carried out in multiple species including *Arabidopsis* and tobacco [5–8], *in planta* cellulase expression would be more advantageous if the enzymes were produced in the lignocellulosic feedstock itself. This would allow the biomass feedstock to serve a dual role, as both enzyme supplier and substrate for ethanol production [9]. One of the challenges associated with expressing hydrolytic enzymes in plants is the prevention of detrimental effects of the enzymes on the plant host. Stunted growth, abnormal stem development, infertility, and depigmentation can result from disruption of host cell structure and function by *in planta* expression of hydrolases [10–13]. A potential solution to this challenge is the expression of hyperthermophilic cellulases that display low enzyme activity at ambient temperatures.

Previous studies have successfully expressed a thermophilic endoglucanase from *Acidothermus cellulolyticus* with an optimum temperature of 80 °C [14] in various plant species without negative effects on plant growth [7, 15, 16]. *In planta* expression of hyperthermophilic cellulases is considered key for simultaneous processing with mild pretreatments (90–120 °C) and enzymatic hydrolysis in one reactor [17]. To achieve this goal, enzymes expressed in the plant should survive and be activated at temperatures over 90 °C. To date, few studies have produced cellulases with optimal temperature over 90 °C *in planta,* and this work has been carried out only in model species including *Arabidopsis* and tobacco [18–20]. Hyperthermophilic endoglucanases have been identified from multiple organisms [21]. Among them, an endoglucanase from *Thermotoga neapolitana* (*Tn*CelB) has an optimal temperature of 106 °C [22], and is one of few characterized endoglucanases which retain activity over 100 °C.

Beyond the original goal of utilizing the plant as a biofactory for enzyme production, *in planta* expression of hydrolytic enzymes has been reported to modify cell wall structure [23–26]. In most previous studies, *in planta* expression of hydrolytic enzymes resulted in a more easily digestible cell wall and enhanced enzymatic saccharification. *In planta* expression of hyperthermophilic cellulases is expected to not only prevent destructive effects of the enzyme on plant host structure and function, but more importantly, it will allow autohydrolysis of feedstock post-harvest. Heat-activated hyperthermophilic endoglucanases in *Arabidopsis* hydrolyzed cellulose in plant hosts and released a small amount of sugar [20]. Higher glucan conversion rates were seen in transgenic *Arabidopsis* overexpressing a hyperthermophilic endoglucanase after enzyme activation, and improved glucan conversion from transgenic plants was suggested to result from more irregular surfaces and empty regions in the plant cell wall caused by endoglucanase activity [24]. This suggests that heat activation of hyperthermophilic enzymes could allow cell wall components to be hydrolyzed or modified by *in planta* produced enzymes prior to the start of processing. In addition, reduced cell wall recalcitrance was reported from endoglucanase overexpressing tobacco and maize even without the enzyme being activated [23]. The previous studies separately investigated effects of *in planta* hyperthermophilic endoglucanase on the plant cell wall with or without the enzyme being activated; however, there was not a systematic comparison of the same tissues with both activation and no activation. Moreover, the majority of the previous research has looked at *Arabidopsis* and tobacco which, while useful model species for biofuels research, are not potential biofuels feedstocks [27]. Therefore, interactions of *in planta* cellulase expression and activation within the plant cell wall in woody plants remains to be investigated.

*Tn*CelB was overexpressed in hybrid poplar (*Populus alba *× *grandidentata*) under the control of the Cauliflower Mosaic Virus 35S promoter. Here, we explore the effect of *in planta Tn*CelB expression on cell wall composition and structure, and the resultant impact on saccharification efficiency both before and after the heat activation of this hydrolytic enzyme.

## Methods

### Synthesis of *Tn*CelB-coding sequence

A hyperthermophilic endoglucanase from *T. neapolitana*, *Tn*CelB (GenBank: AAC95060.1) was codon optimized based on the codon usage of *Populus trichocarpa* using the online tool Optimizer [28]. A synthetic intron was inserted into the DNA sequence [29], and the targeting sequence from tobacco pathogenesis-related protein 1a (PR1a) was used to target *Tn*CelB to apoplast [30]. For convenience of molecular cloning, the second amino acid (asparagine) of the original PR1a was mutated to a glutamate (Additional file 1: Table S1). Codon-optimized *Tn*CelB containing the synthetic intron and the apoplast target sequence was synthesized (GenScript ^®^, Piscataway, NJ, USA). Apoplast-targeted *Tn*CelB was cloned into the *pCambia*1300 binary vector under the control of the Cauliflower Mosaic Virus 35S promoter (CaMV) and confirmed by sequencing. Correct splicing was confirmed in poplar first by PCR comparing the plasmid DNA to transgenic poplar cDNA using primers flanking the synthetic intron (Additional file 1: Fig. S1), and then by sequencing of the amplified cDNA. Apoplast targeting by the mutated PR1a sequence was confirmed by fusing the sequence with GFP and carrying out transient expression in onion epidermal cells (Additional file 1: Fig. S2).

### Plant material

The pCambia 1300 binary vector containing the *Tn*CelB gene driven by the CaMV 35S promoter was transformed into *Agrobacterium tumefaciens* Strain GV3101. *Agrobacterium*-mediated transformation of poplar (*Populus alba *× *grandidentata*) was conducted as previously described [31]. Four transgenic events were confirmed to contain the expression cassette using genomic PCR (Additional file 1: Table S1). Each transgenic event, along with the non-transgenic control (WT), was vegetatively propagated to produce multiple lines (*n* = 6–8) in tissue culture before they were moved to the greenhouse. The plants were grown in the greenhouse under 16 h light (24 °C)/8 h dark (18 °C) and fertilized weekly. Four-month-old plants were measured prior to being destructively harvested for analysis. Plant height was measured from soil to the top most stem, and diameter was measured at 10 cm above the soil using a digital caliper. Leaf number was counted from top to bottom of stems. The growth stage of plant tissue was determined by plastochron index [32]. The first leaf with a blade length larger than 5 cm was assigned as P0 and the leaf immediately below P0 was assigned as P1, then P2, and so on. Internode length was calculated as stem height divided by leaf number. P15 and P16 were collected as leaf tissue for analysis. Stem sections between P10 and P30 were used for the collection of developing xylem. Bark was peeled from the wood, and the outer layer of tissue was scraped from the wood using a scalpel. Wood tissue was collected from P10 to the base for analysis of cell wall composition and enzymatic hydrolysis. Intact stems from P30 to bottom were treated at 100 °C for 4 h to activate *Tn*CelB enzyme to produce heat-treated stems, while stems from P10 to 30 did not undergo heat treatment, but were dried only after the removal of the developing xylem as untreated stems. All stem tissues were debarked and pith removed before chemical analysis.

### Quantitative RT-PCR analysis

Total RNA was extracted from leaf and developing xylem tissue separately based on the previous methods [33]. In short, 0.2 g tissue was ground in liquid nitrogen with 10 ml extraction buffer containing 10 M lithium chloride (LiCl), 0.5 M EDTA, 1 M Tris (pH 8.0), 1.5% lauryl sulfate lithium salt, 1 mM aurintricarboxylic acid, 10 mM dithiothreitol, 5 mM thiourea, 0.1% sodium deoxycholate, 1% tergitol NP-40, and 2% PVPP (w/v). The homogenate was then transferred into a 50 ml Falcon tube and 1/10 volume 3.3 M sodium acetate (pH 6.1) and 1 volume chilled isopropanol added. The mixture was incubated at − 80 °C for at least 30 min followed by centrifugation at 4000 rpm for 40 min. The pellets were resuspended in 2 ml TE and 2 ml 5 M NaCl, 1 ml of 10% CTAB added, and incubated at 65 °C for 5 min. An equal volume of chloroform: IAA was added, followed by centrifugation at 4000 rpm for 20 min. The aqueous phase was transferred to a new falcon tube and ¼ volume of 10 M LiCl added prior to overnight incubation at − 20 °C. The mixture was centrifuged and all remaining liquid removed. The pellet was dissolved in 900 µl TE buffer and 900 µl chilled isopropanol and 3.3 M sodium acetate (pH 8.0) added to precipitate nucleotides, followed by centrifugation at 14,000 rpm for 60 min. The remaining pellet was washed with 70% ethanol and then dried at room temperature. Dried pellets were dissolved in 50 µl autoclaved DEPC-treated water. Quality and quantity of extracted RNA were assessed by NanoDrop (Thermo Fisher Scientific, Waltham, MA, USA), as well as agarose gel electrophoresis.

RNA was treated with DNase according to manufacturer’s instructions of RQ1 RNase-Free DNase (Promega, Madison, WI, USA). DNase-treated RNA was used as a template for cDNA synthesis using High-Capacity cDNA Reverse Transcription Kits (Applied Biosystems, Foster city, CA, USA). For qPCR analysis, PCR reaction was conducted using PerfeCTa SYBR^®^ Green FastMix (Quantabio, Beverly, MA, USA). Polyubiquitin (UBQ11, GeneBank No. BU879229) and elongation factor 1-beta 2 (EF1β, Gene Symbol: LOC7489318) were used as a reference genes [34]. The primers for all genes of interest and housekeeping genes are listed in Additional file 1: Table S1. *Tn*CelB transcript level was quantified using delta critical threshold values (ΔCt) calculating as ΔCt = 2 ^−(Ct *Tn*CelB − Ct reference genes)^ [35].

### Enzymatic activity

Enzymatic activity of *in planta Tn*CelB was measured by 4-methylumbelliferone β-d-cellobioside (MUC) assay and carboxymethyl cellulose (CMC) assay. Total protein was extracted from leaf and developing xylem tissue based on the protocol by Côté and Rutledge [36]. Protein concentration was measured by Bradford Reagent (Sigma-Aldrich, St. Louis, MO, USA). Endoglucanase activity of *Tn*CelB was assessed using MUC (Sigma-Aldrich, St. Louis, MO, USA) as substrate [37]. Total soluble protein (TSP) extracted from 50 mg ground tissue was used for enzymatic assay as described by Ransom et al. [38]. 5 µl TSP (representing 0.1–0.3 μg TSP) was added into a reaction well in a 96-well plate with 25 µl substrate solution. To assess the temperature optimum, the reaction was conducted at multiple temperatures including 25, 35, 45, 55, 65, 75, 85, 95, and 100 °C. The reaction was stopped by addition of stop solution (0.36 M Na_2_CO_3_) at 0, 10, 20, and 30 min, respectively. Production of 4-methylumbelliferone (4Mu) was measured by fluorescence at 465 nm with an excitation wavelength of 360 nm using a Synergy H1™ microplate reader (BioTek, Winooski, VT, USA). Readings were conducted in triplicate. Enzymatic activity from transgenic events was calculated as the reaction rate (µM4Mu/0.1 μg TSP/min) over the 30 min reaction period.

CMC assay used on 1% (w/v) carboxymethylcellulose sodium salt in 50 mM sodium citrate (pH 5.0) as the substrate. 100 µl TSP (representing 2–6 µg TSP) was added to test tubes with 500 µl 1% (w/v) CMC substrate and 400 µl sodium citrate buffer. The reactions were conducted at 100 °C for 1 h and released reducing sugar was measure by 3,5-dinitrosalicylic acid (DNS) assay along with glucose standards [39]. 3 ml DNS reagent was reacted with 1 ml reaction mixture or sugar standards and incubated at 90 °C for 5 min. Concentration of reducing sugar was measure by absorbance at 575 nm using a Synergy H1™ microplate reader (BioTek, Winooski, VT, USA). One unit of enzyme activity (U) is the amount required to release 1 mg/ml of reducing sugar per hour at 100 °C.

Because heat could activate TnCelB, instead of normalizing activities using heat-inactivated TSP from the same line, non-transgenic WT control was used to normalize activities of transgenic lines as described previously [20].

### Cell wall composition

Stem tissue from three plants per event was analyzed for cell wall composition. Air-dried stem tissue was ground by Willey mill to pass through a 40-mesh filter. Ground stem tissue underwent acetone extraction for 8 h in a soxhlet extractor. Acetone extractives were measured gravimetrically using pre-extraction and post-extraction sample weights. Lignin and structural carbohydrates in extractive-free tissue were quantified by acid hydrolysis [40]. Briefly, 3 ml of 72% sulfuric acid was added to 200 mg oven-dried tissue in a test tube. The reaction was mixed for 30 s every 10 min for 2 h. An addition of 112 ml distilled water diluted the sulfuric acid to concentration of 4% and was reacted at 120 °C for 1 h. The liquid hydrolysate was collected by vacuum-filtering through dry, pre-weighed medium coarseness crucibles and ion-exchange chromatography (Dionex ICS-3000, Thermo Fisher Scientific, Waltham, MA, USA) equipped with PA1 Analytical & Guard Columns (Thermo Fisher Scientific) was used to quantify structural carbohydrates. Water was used as the carrier at a rate of 1 ml/min with a post-column addition of 200 mM sodium hydroxide. Acid-soluble lignin was determined by reading absorbance at 205 nm on SmartSpec Plus spectrophotometer (Bio-Rad, Herculus, CA, USA), and acid-insoluble lignin was measured gravimetrically [40].

### Crystallinity of cellulose

Cellulose crystallinity index (CI) was measure by X-ray diffraction (XRD) at Cornell High-Energy Synchrotron Source with a beamline energy of 19.3 keV (equivalent to a wavelength of 0.63 Å). A high-flux beam was focus on a 10 µm-diameter spot and accepted by a 4 mrad polycapillary optic. Ground stem tissue was packed in an aluminum plate with multiple wells of 6.35 mm diameter and 4.78 mm depth. Signals were detected by ADSC Quantum-210 placed at 280 mm from the samples. Data were integrated by ADX data collection GUI. CI was calculated as *I*_002_ − *I*_am_/*I*_002_ × 100, where *I*_002_ or *I*_am_ was the peak height above baseline at 22.5° or 18°, respectively [41].

### Pretreatment

Ground wood tissue was pretreated with 1% sulfuric acid at a ratio of 10:1 at 120 °C for 30 min [42]. Pretreated biomass was washed with distilled water (3 × 50 ml) and dried at 60 °C overnight. Carbohydrate release by pretreatment was assessed in the hydrolysate by IC as described above for structural carbohydrates.

### Enzymatic hydrolysis

Enzymatic hydrolysis was carried out as described by Resch et al. [43]. 14 mg of biomass was loaded into 1.4 ml 50 mM citrate buffer solution (pH 4.8) containing Accellerase^®^1500 and Accellerase^®^ XY (Dupont); Accellerase^®^1500 at 20 FPU/mg glucan and Accellerase^®^ XY at 0.1 ml/g dry biomass. Enzymatic hydrolysis was conducted at 37 °C at 200 rpm. Liquid hydrolysate was collected at 0, 3, 6, 12, 24, 48, 72, 96, and 120 h, and enzymes inactivated by heat treatment at 100 °C for 10 min. Released glucose was quantified with a Dionex ICS-3000 ion-exchange chromatography system, as described above.

### Statistical analysis

All events (6–8 plants per event) are included in phenotypic growth analysis. Tissue from three plants per event was used for all laboratory analysis. Technical replicates were carried out. Statistical significance was analyzed using one-way ANOVA followed by Tukey post hoc analysis in R [44].

## Results

### Expression and activity of *TnCelB* in transgenic events

Four confirmed transgenic events containing the codon-optimized *TnCelB* gene driven by the Cauliflower Mosaic Virus 35S promoter (CaMV 35S) and targeted to the apoplast were generated. Each event was vegetatively propagated to produce multiple plants and grown in the greenhouse for 4 months before being harvested for analysis.

**Fig. 1:**
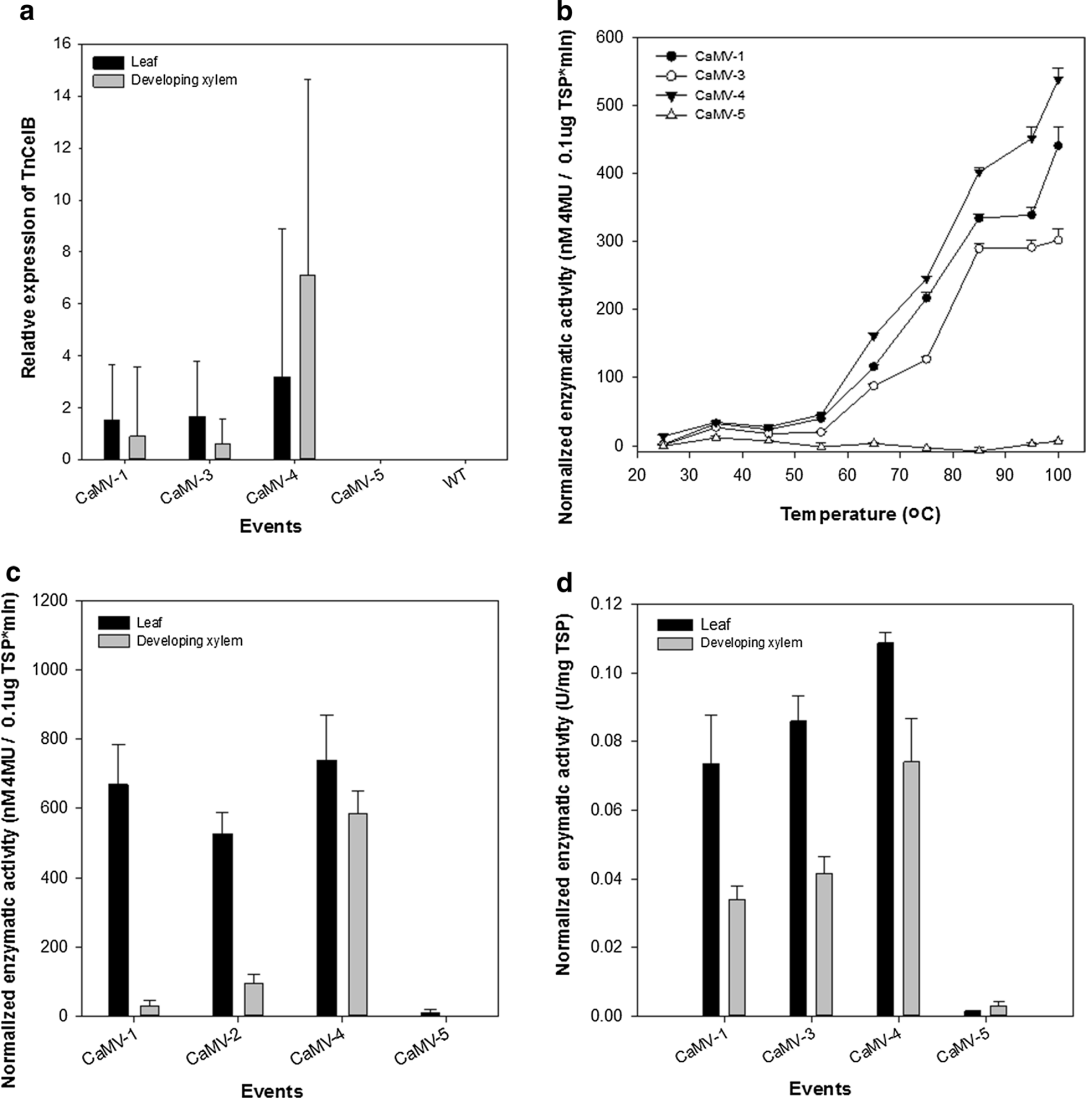
Expression of *Tn*CelB in transgenic poplar lines. **a** Relative transcript level of *TnCelB* in leaf and developing xylem in transgenic events and WT measured by qPCR using reference genes (UBQ11 and EF1β) as the internal control; **b** MUCase activities of total soluble protein (TSP) from leaf tissue of transgenic events over a temperature range (25–100 °C); **c** MUCase activity of TSP extracted from leaf tissue and developing xylem of transgenic events and WT at 100 °C; and **d** CMCase activity of TSP extracted from leaf tissue and developing xylem of transgenic events and WT at 100 °C. The enzymatic activities of transgenic events were normalized to WT levels. Each value represents mean of technical triplicates of three individual plants per event with the standard error

Quantitative RT-PCR results showed that three of the four *Tn*CelB overexpression lines have *Tn*CelB transcripts accumulating in both leaf and developing xylem, with CaMV-4 having the highest expression in both tissue types. Expression levels seen in CaMV-5 were similar to wild-type (WT) levels (Fig. 1a).

Initially, TSP from leaf tissue of four transgenic events was used to assess activity of TnCelB over a temperature gradient (25–100 °C) by MUC assay to confirm the temperature optimum of the codon optimized, plant-produced enzyme. The activity of plant-produced *Tn*CelB from the transgenic events was calculated and normalized according to the previous research using activity from WT as a baseline [20]. Activity of the plant-made endoglucanase increased along a temperature gradient and reached the highest activity at 100 °C, although it appears that activity may continue to increase beyond this temperature (Fig. 1b). CaMV-4 transgenic event showed endoglucanase activity starting from 25 °C (average of 13.7 Units in CaMV-4 at 25 °C after normalization to WT), indicating that *in planta* TnCelB at high expression levels had detectable, albeit low levels of hydrolytic activity at ambient temperatures (Fig. 1b). TnCelB activity was quantified by MUC assay at 100 °C. Enzymatic activities from transgenic events are consistent with *TnCelB* transcripts across events (Fig. 1c). CaMV-4 has the most abundant *TnCelB* transcripts and the highest MUCase activity in both leaf and developing xylem. Consistent with qRT-PCR results, CaMV-5 had imperceptible activity along the temperature range (Fig. 1c).

Enzymatic activity of *in planta Tn*CelB towards cellulosic substrates at 100 °C were quantified by CMC assay and released reducing sugar was measured by DNS assay. The results showed that TSP from leaf and developing xylem of CaMV-4 had the highest hydrolytic activity on CMC, followed by CaMV-3 and CaMV-1 (Fig. 1d). Similar to the MUC assay results, CaMV-5 had extremely low activity towards CMC (Fig. 1d).

### Phenotype of *Tn*CelB-overexpressing transgenic plants

**Fig. 2:**
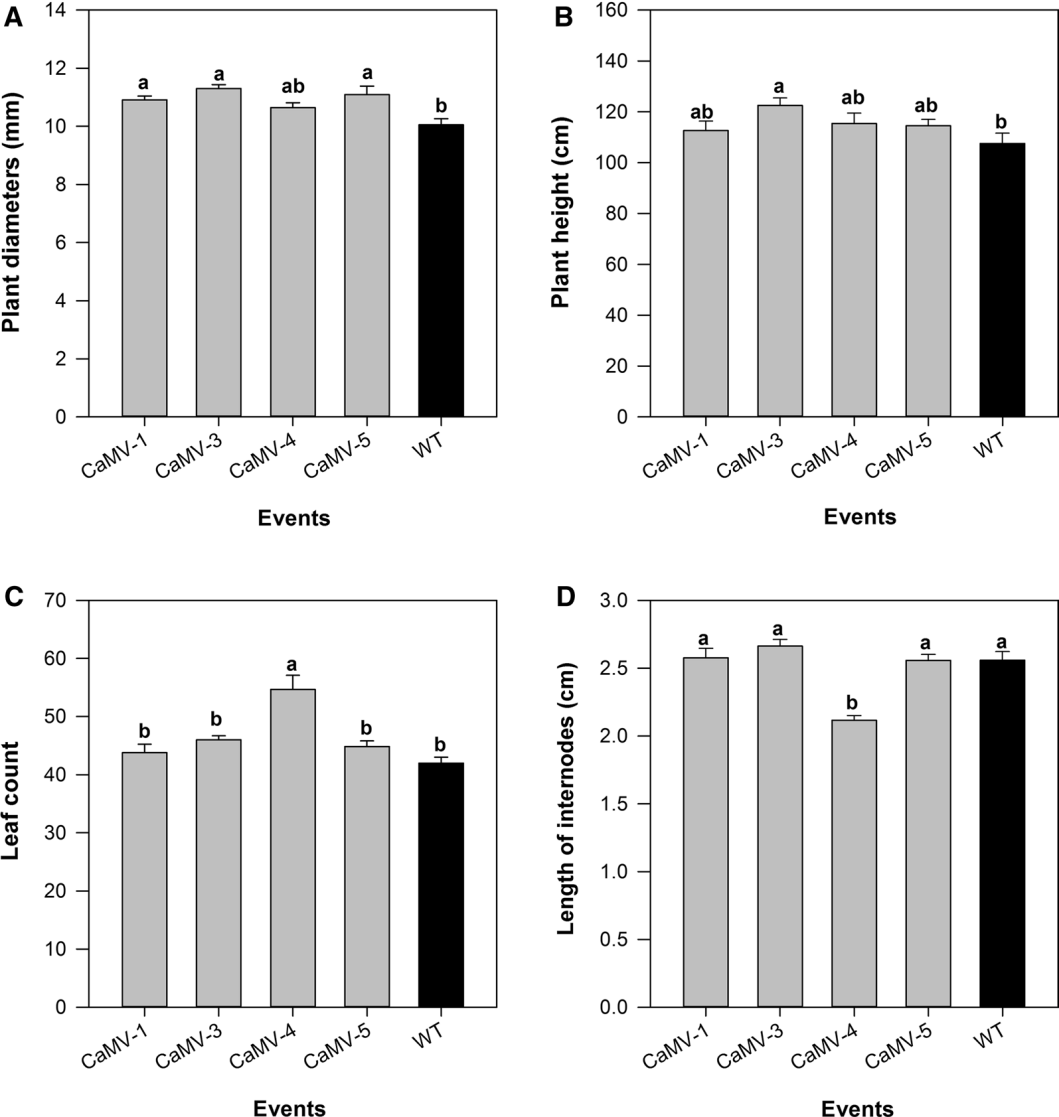
Growth comparison of 4-month-old greenhouse-grown transgenic *Tn*CelB-overexpressing poplar and WT control. **A** Stem diameter; **B** plant height; **C** leaf number; and **D** length of internode. Each bar represents mean of five individual plants. Different letters represent significant differences among the samples (*p* ≤ 0.05) as calculated by one-way ANOVA followed by Tukey post hoc analysis

Phenotypic measurements including height, stem diameter, number of leaves (internodes), and average length of internode were assessed at the time of harvest. Three transgenic events (CaMV-1, CaMV-3, and CaMV-5) had increased stem diameters relative to WT (Fig. 2A) and one transgenic event (CaMV-3) was significantly taller than WT (Fig. 2B). One event, CaMV-4, had increased total number of internodes and produced more leaves during growth (Fig. 2C). The change in internode number was offset by a decrease in the average length of internodes (Fig. 2D), and therefore, there was no change in overall height. CaMV-4 displayed a wrinkled leaf phenotype that was present from tissue culture through greenhouse growth (Additional file 1: Fig. S3a). This line also displayed an abnormal phenotype in the greenhouse, including showing minor signs of dehydration at the time of harvest (Additional file 1: Fig. S3b).

### Cell wall composition and cellulose structure

**Fig. 3:**
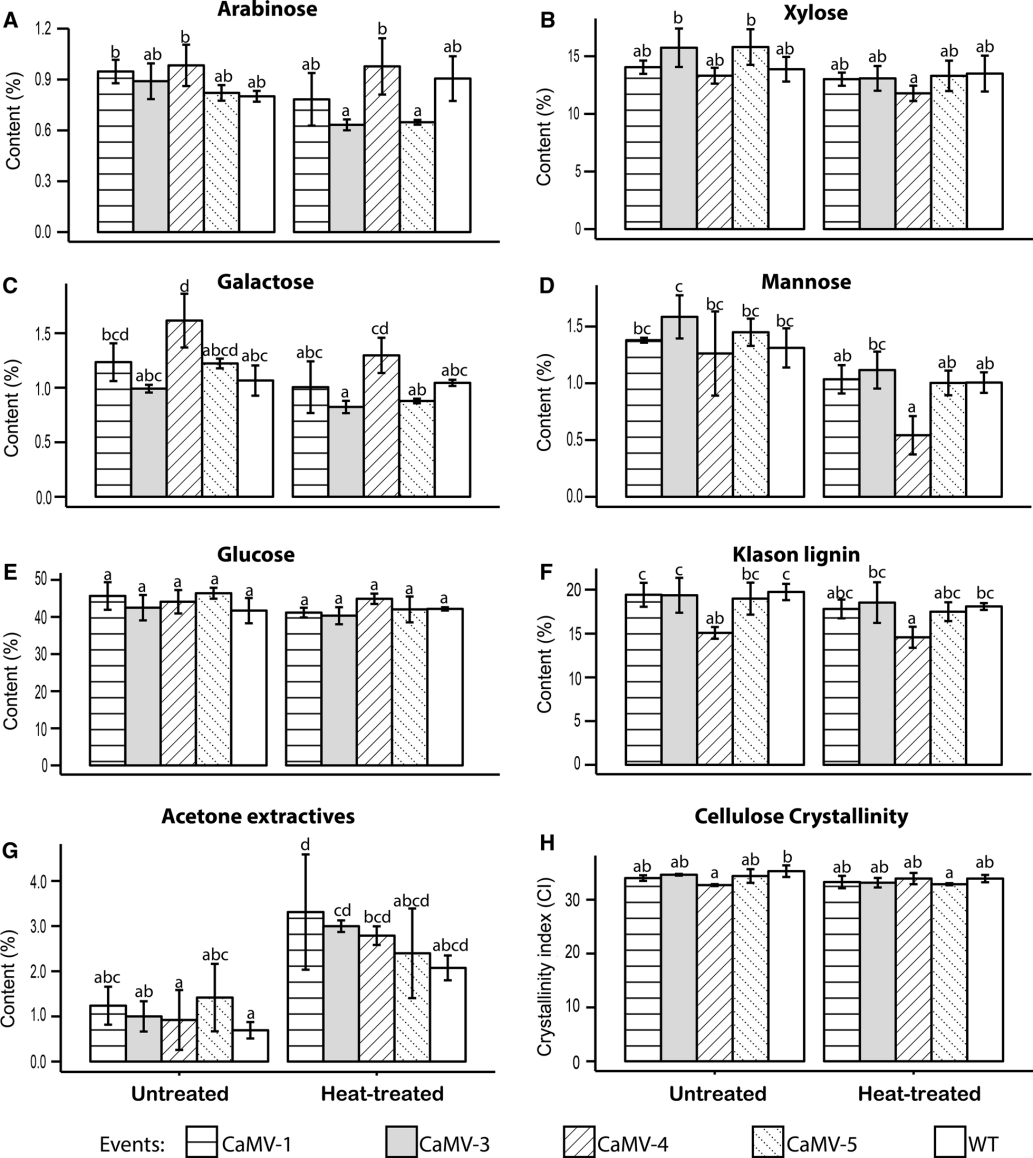
Comparison of cell wall composition and cellulose crystallinity of untreated and heat-treated wood for each event. Structural sugar monomers including **A** arabinose; **B** xylose; **C** galactose; **D** mannose; **E** glucose; and **F** Klason lignin; **G** acetone extractives; and **H** cellulose crystallinity index of untreated and heat-treated stem in each event. Each bar represents mean of technical duplicates of three individual plants per event with the standard error. Different letters represent significant differences among the samples (*p* ≤ 0.05) as calculated by one-way ANOVA followed by Tukey post hoc analysis

To investigate the effect of *in planta Tn*CelB expression on the cell wall structure before and after enzymes were activated, stems were divided into two sections. Stem sections from P10 to P30 were used as is, with no heat treatment applied before removal of bark. Stem sections from P30 to the base were heat-treated with bark still on at 100 °C for 4 h immediately after harvest. Following heat treatment bark and pith were removed. Cell wall structural components were assessed in acetone-extracted untreated and heat-treated stem tissue of transgenic and WT poplar. Carbohydrates in untreated and heat-treated transgenic lines were not significantly different compared to WT except untreated CaMV-4 had increased galactose content (Fig. 3A–E). Besides minor changes in carbohydrates, CaMV-4 had significantly decreased acid-insoluble lignin relative to WT in both untreated and heat-treated stems (Fig. 3F).

Statistical differences were absent in most pairwise comparisons of individual carbohydrates between untreated and heat-treated stems (Fig. 3A–E), except heat-treated CaMV-4 had significantly reduced mannose compared that of untreated stem (Fig. 3D). Statistically increased levels of acetone extractives were present in heat-treated wood of CaMV-1, CaMV-3, and CaMV-4 relative to untreated stem of corresponding event, although a similar trend also existed in CaMV-5 and WT (Fig. 2G). Acetone extractives contain phenolic substances and sugars that are not part of the structural cell wall [45]. Heat has been found to degrade hemicellulose but not lignin in poplar [46]; therefore, increased acetone extractives in heat-treated stems likely contained sugars derived from hemicellulose.

To assess whether changes in cell wall composition were the result of changes in cell wall formation or due to the action of the transgene, we carried out qRT-PCR on a select number of secondary cell wall formation related genes. Expression levels of genes involved in cellulose (cellulose synthase; *CesA4*, *CesA7*, *CesA8*, and Korrigan), monolignol (Cinnamyl-alcohol dehydrogenase; *CAD*), and hemicellulose (glycosyltransferase *GT47C*) biosynthesis in the secondary cell wall were assessed. There was no difference in expression of these genes between transgenic events and WT (Additional file 1: Fig. S4).

Cellulose crystallinity index from untreated and heat-treated stem sections was determined by X-ray diffraction. Crystallinity index was calculated as the ratio of crystalline cellulose to non-crystalline cellulose [47]. CaMV-4 is the only event that displayed significantly reduced CI relative to the WT control, suggesting that the high expression of recombinant endoglucanase had an effect on cellulose structure even without heat induction (Fig. 3H). However, the difference of comparison between CaMV-4 and WT disappeared in heat-treated stems (Fig. 3H).

### Pretreatment and enzymatic hydrolysis

**Fig. 4:**
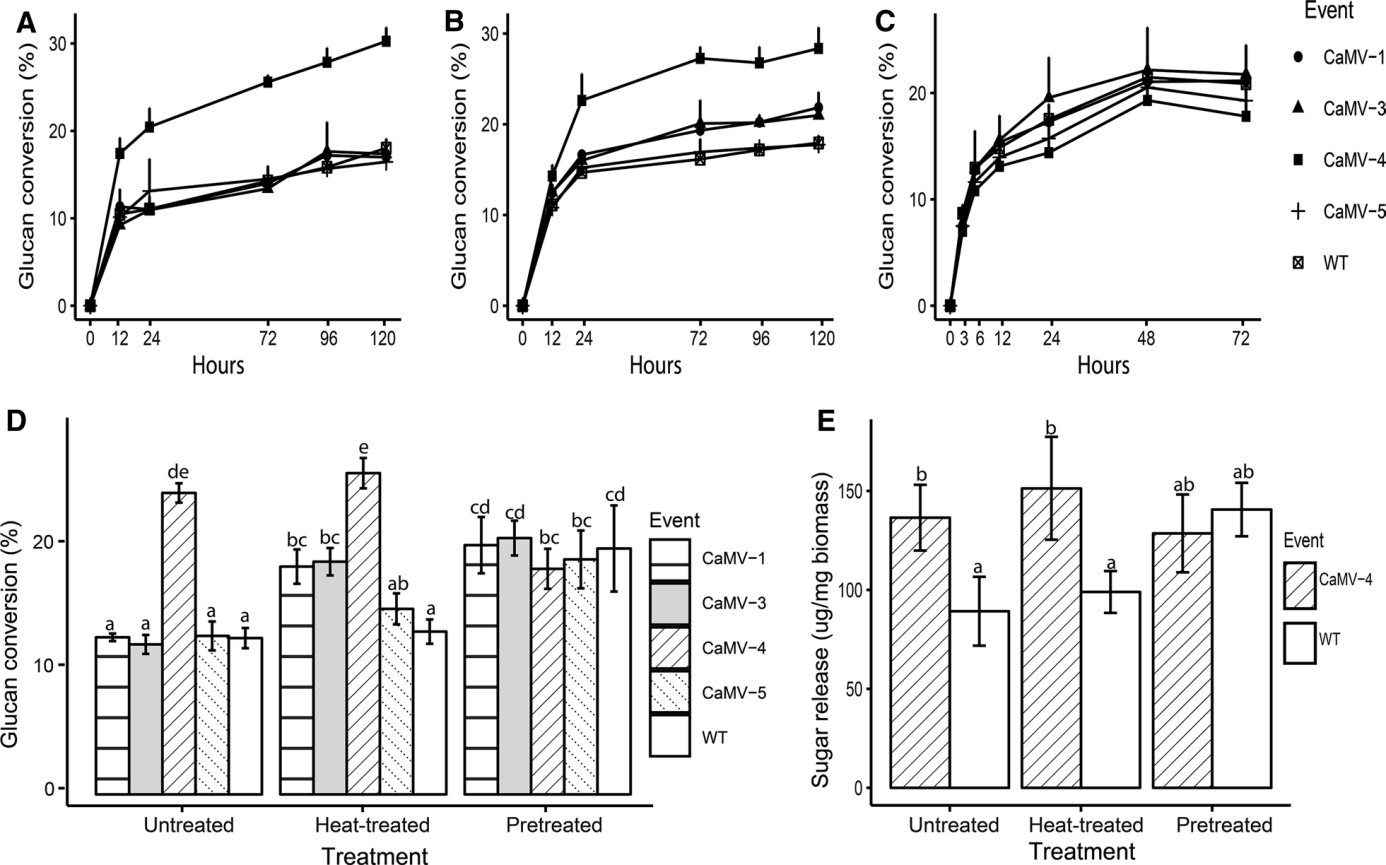
Enzymatic saccharification efficiency of transgenic events and WT. Glucan conversion rates of untreated biomass (**A**), heat-treated biomass (**B**), and heat-treated biomass followed by a mild acid pretreatment (**C**). Liquid hydrolysate was collected at different timepoints over 72–120 h, and glucose measured by ion-exchange chromatography. **D** Comparison of glucan conversion rates at 72 h of untreated biomass, heat-treated biomass, and pretreated biomass of transgenic events and WT. **E** Comparison of released glucose concentration of CaMV-4 and WT among three treatments. Each bar represents mean of three individual plants per event with the standard error. Different letters represent significant differences among the samples (*p* ≤ 0.05) as calculated by one-way ANOVA followed by Tukey post hoc analysis

Untreated and heat-treated stem sections underwent enzymatic hydrolysis using Accellerase^®^1500 and Accellerase^®^ XY (Dupont) to determine if glucose release was improved by gene expression and enzyme activation. Released glucose was determined at multiple timepoints up to 120 h. For untreated stems, higher glucan conversion rates were observed exclusively in CaMV-4 lines relative to WT (Fig. 4A, D). Heat-treated stems of CaMV-1, CaMV-3, and CaMV-4 had higher glucan conversion rates than WT, and CaMV-4 still had the highest glucan conversation rates among all the events (Fig. 4B, D).

To further assess the potential of these lines, heat-treated stem tissue underwent a mild acid pretreatment followed by enzymatic hydrolysis for 72 h when glucan conversation rates reached a plateau. Interestingly, CaMV-4 had similar glucan conversion to WT (Fig. 4C, D) following pretreatment. To compare saccharification efficiencies across events and treatments, glucan conversion rates of all stem samples at 72 h were selected for data analysis (Fig. 4D). CaMV-1 and CaMV-3 had significantly increased glucan conversion rates in heat-treated stem sections relative to corresponding untreated sections, suggesting that activation of *in planta* expressed *Tn*CelB enhanced saccharification efficiency (Fig. 4D). These differences were not seen in CaMV-5 or WT, indicating that the heat treatment itself had negligible effect on saccharification. These differences were also not detected in CaMV-4, which had highest saccharification rates in both untreated and heat-treated stem sections. It is possible that CaMV-4 had relatively more easily digestible cell wall in untreated stems, as suggested by reduced lignin and cellulose crystallinity, and as a result, activated *in planta* expressed *Tn*CelB was no longer a major factor in enhancing hydrolysis in this line.

Comparing sugar release between WT and CaMV-4 across the three treatments, pretreated WT stem sections released more glucose than unpretreated WT as expected (Fig. 4E). Unpretreated CaMV-4 tissue, including untreated and heat-treated stem sections, released similar glucose to pretreated CaMV-4 tissue. In addition, unpretreated CaMV-4 had a comparable glucose release level to pretreated WT during enzymatic saccharification (Fig. 4E).

Pretreatment liquid was assessed for sugar concentration. The results showed carbohydrate monomers consistent with hemicellulose degradation in all lines including WT (Table 1). CaMV-4 released almost twice as much glucose relative to WT during pretreatment (Table 1), suggesting that a larger portion of cellulose was degraded during pretreatment in CaMV-4 relative to WT. Higher glucose release during pretreatment of CaMV-4 indicated that the plants had more digestible cellulose structure due to expression and activation of *in planta Tn*CelB. It is possible that digestibility of the remaining cellulose portion in CaMV-4 following pretreatment was similar to that remaining in the WT, resulting in similar efficiency of enzymatic hydrolysis.

**Table 1:** Carbohydrate content in pretreatment hydrolysate (% biomass)

Event	Arabinose	Galactose	Glucose	Xylose	Mannose
CaMV-1	0.83 ± 0.13	1.04 ± 0.13	5.37 ± 0.76	9.92 ± 0.30	0.61 ± 0.02
CaMV-3	0.67 ± 0.03	0.82 ± 0.03	4.61 ± 0.58	9.19 ± 0.66	0.60 ± 0.09
CaMV-4	0.97 ± 0.06	1.32 ± 0.11	9.22 ± 0.33**	8.56 ± 0.20	0.16 ± 0.02**
CaMV-5	0.71 ± 0.04	0.93 ± 0.08	6.5 ± 0.21	10.01 ± 0.05*	0.52 ± 0.05
WT	0.90 ± 0.05	0.98 ± 0.08	5.00 ± 1.01	9.22 ± 0.18	0.46 ± 0.07

Asterisks indicate significant differences in comparison with WT. *p < 0.05, **p<0.01 (one-way ANOVA), *n* = 3

## Discussion

*In planta* production of cell wall degrading enzymes in lignocellulosic biomass could benefit both enzyme production and polysaccharide conversion from plant biomass [9, 48]. Besides preventing detrimental effects on plant hosts, expression of hyperthermophilic cellulases with optimal temperatures over 90 °C in feedstocks could allow simultaneous processing, combining pretreatment and enzymatic hydrolysis without additive cellulase enzymes. Hyperthermophilic cellulases, those with optimal temperatures of higher than 80 °C, have thus far been expressed only in tobacco and *Arabidopsis* [18, 20], which are not potential dedicated biofuels feedstocks. Expression of hyperthermophilic cellulases in feedstocks is necessary to optimize the process of ethanol production from lignocellulosic biomass. This is particularly important to address in woody feedstocks, which are significantly different from current model plants.

Here, we expressed a hyperthermophilic endoglucanase in poplar, a promising feedstock for biofuels [49], and investigated the effect of *in planta* endoglucanase production on plant growth, cell wall composition and structure, and saccharification efficiency. We found that transgenic poplar overexpressing *Tn*CelB had improved saccharification rates, which will benefit ethanol production.

### Poplar-derived *Tn*CelB retains thermoactivity

An endoglucanase from *T. neapolitana* (*Tn*CelB) was selected for this study, based on its high optimal temperature of 106 °C [22], and targeted to the apoplast. The transgenic plants generated in this study successfully produced *Tn*CelB protein with increasing activity up to 100 °C. This is the first study expressing hyperthermophilic cellulases with high activity at 100 °C in a woody plant species and this work further confirmed the capability of plant-based protein production platform to keep thermophilic characteristics of original proteins [48].

According to the previous research, *Tn*CelB was only activated at temperatures over 80 °C [22]. However, here, we detected weak activity of poplar-derived *Tn*CelB at temperatures over 25 °C in the highest expression event (Fig. 1b). This suggests that this codon-optimized TnCelB could be active over a large temperature range. The weak hydrolytic activity at ambient temperature conceivably causes changes in phenotype and cell wall composition.

Reducing sugar released from CMC treated by TSP of transgenic events (Fig. 1d) indicated that *in planta Tn*CelB obtained endoglucanase activity of cleaving celluloses, and suggested that plant-produced *Tn*CelB without purification could be a supplement to cellulase cocktails.

### Overexpression of *Tn*CelB in poplar altered plant phenotypes

Previously, a variety of cellulases have been overexpressed in a different plant species, most of which were annual plants or grasses including *Arabidopsis*, tobacco, alfalfa, and maize [7, 38, 50]. Some studies have illustrated that *in planta* expression of cell wall degrading enzymes altered plant cell wall composition and structure and resulted in more digestible cell walls, which suggested that *in planta* enzymes interfere with cell wall synthesis [25, 51–54]. In this study, transgenic poplar overexpressing *Tn*CelB had altered growth parameters from WT with one event having increased plant height and three transgenic events with increased stem diameters compared to the non-transgenic control (Fig. 2). Since CaMV-5, a transgenic event without *Tn*CelB transcripts or activity, also had increased stem diameter, it is likely that this phenotypic change is due to insertional effects. Improved plant height or stem diameter has not previously been reported in plants expressing microorganism-originated cellulases. However, *in planta* expression of plant endoglucanases has resulted in altered plant biomass. For example, increased biomass production has been observed in transgenic poplar overexpressing an *Arabidopsis* endoglucanase [55] and transgenic poplar lines, with RNAi down-regulated *Korrigan,* showed reduced growth [56].

CaMV-4 events with the highest *Tn*CelB expression had a similar wrinkled leaf phenotype to transgenic tobacco expressing a mesophilic *Trichoderma reesei* endoglucanase [52]. It is possible that the relatively high expression of *Tn*CelB in leaves in this line could interact with cellulose in the primary cell wall during plant growth because of higher accessibility to cellulose compared to in the secondary cell wall [57]. In addition, the slight dehydration of the apical stems of CaMV-4 events was seen when plants reached approximately 1.1 m in height, which could suggest disruptive effects of high *Tn*CelB expression on xylem tissue resulting in malfunction of water transportation, but this was not confirmed experimentally. Interestingly, shorter internodes and increased number of internodes were only observed in CaMV-4, the line with the highest expression and activity. These phenotypic alterations in CaMV-4 could due to abundant *Tn*CelB accumulated at the apoplast. A previous study reported that decreased plant growth and reduced structural glucose contents were found in the transgenic tobacco with apoplast-targeted mesophilic endoglucanases but not in lines with the enzymes targeted to the endoplasmic reticulum [52]. Therefore, hydrolytic enzymes accumulating in the apoplast could cause negative phenotypic changes. The negative phenotypic changes in CaMV-4 could be due to abundant *Tn*CelB accumulating in the apoplast.

### Post-harvest activation of *Tn*CelB results in altered cell wall composition

Prior to heat treatment, *Tn*CelB-overexpressing transgenic lines displayed only minor differences in carbohydrate composition compared to WT (Fig. 3A–E). The event with the highest *Tn*CelB expression, CaMV-4, had significantly decreased lignin content relative to WT (Fig. 3F). Interestingly, reduced lignin contents have also been reported from transgenic switchgrass expressing beetle-derived endoglucanase [58]. Genes involved in cellulose, monolignol, and hemicellulose synthesis did not display altered expression in the *Tn*CelB-overexpressing transgenic plants, indicating that the alteration of cell wall composition in transgenic events does not appear to be due to changes in expression of cell wall component biosynthesis genes. Reduced lignin contents in CaMV-4 events might be explained by abundant endoglucanase accumulated at the apoplast interfering with lignin deposition, since cellulase–lignin interactions have been identified in previous studies [59, 60]. Interestingly increased lignin content was seen in *Korrigan* RNAi transgenic poplar [56], which also suggested that *in planta* endoglucanases may interact with lignin synthesis (including polymerization and deposition), especially endoglucanases located close to the cell wall. CaMV-4 had reduced cellulose crystallinity in untreated stem sections (Fig. 3E), which was also seen in transgenic *Arabidopsis* overexpressing aspen endoglucanases [61] and transgenic poplar expressing *Arabidopsis* endoglucanase [56].

Comparing between untreated and heat-treatment stems across individual events, both transgenic events and WT shared the same pattern that acetone extractive content was higher in heat-treated stems compared to untreated stems (Fig. 3G). The previous research showed that heat could degrade hemicellulose but not cellulose or lignin [45]. Therefore, increased acetone extractives in heat-treated stem most likely contained degraded hemicellulose. Although statistical differences across untreated and corresponding heat-treated stems were absent in most individual sugar monomers of hemicellulose (arabinose, galactose, glucose, xylose, and mannose), there is an apparent trend that heat-treated stems had decreased mannose relative to untreated stems (Fig. 3D), indicating that mannose-associated polymers were likely partially degraded by heat.

The highest expressing event, CaMV-4, was the only event showing reduced cellulose crystallinity in untreated wood compared to WT (Fig. 3H). The difference was absent in comparison between heat-treated CaMV-4 and WT due to relatively higher CI in heat-treated CaMV-4 (Fig. 3H). The higher CI in CaMV-4 after heat treatment could be due to the efficient hydrolysis of amorphous cellulose by active *Tn*CelB [62], making the increased cellulose crystallinity index the direct result of a decrease in amorphous cellulose.

*In planta* expressed *Tn*CelB modified secondary cell wall composition and structure, especially when *Tn*CelB was expressed at an elevated level in the developing xylem. The changes include reduced lignin and lower cellulose crystallinity, both characteristics associated with a more digestible cell wall.

### TnCelB overexpression lines have improved saccharification efficiency

Only the transgenic line with the highest expression of *Tn*CelB in the xylem (CaMV-4) showed increased glucan conversion rates compared to WT when no treatment was applied (Fig. 4A). The enhanced saccharification rates were possibly the result of more easily digestible cell wall structure as indicated by reduced lignin and decreased cellulose crystallinity. As expected, after activation by heat, stems of WT and CaMV-5 with undetectable *Tn*CelB expression showed no difference in cell wall composition compared to untreated stems (Fig. 4D), since heat treatment alone has little impact on lignin and cellulose in wood [46]. Transgenic events with moderate *Tn*CelB expression levels (CaMV-1 and CaMV-3) had increased conversion rates relative to untreated stems (Fig. 4D). The enhanced saccharification of CaMV-1 and CaMV-3 after heat treatment could be due to activated endoglucanase activity from *Tn*CelB at the apoplast towards glucan in the cell wall. In addition, transgenic poplar with moderate to high *Tn*CelB expression level (CaMV-1, CaMV-3, and CaMV-4) had higher glucan conversion rates than the WT and the transgenic line that showed no expression (Fig. 4D). The result suggested that for those transgenic events with modest *Tn*CelB expression but without drastically altered cell wall composition, activation of the *in planta* expressed endoglucanase increased cellulose saccharification. For CaMV-4, with the highest *Tn*CelB expression and modified cell wall structure, *in planta* heat-activated *Tn*CelB did not enhance glucan conversion rates, which suggested that activation of *Tn*CelB in CaMV-4 was not a major factor for improved saccharification efficiency. However, the benefit of elevated expression level in CaMV-4 is demonstrated earlier in that this line does not require pretreatment to release equivalent levels of glucose to pretreated WT biomass (Fig. 4E).

**Fig. 5:**
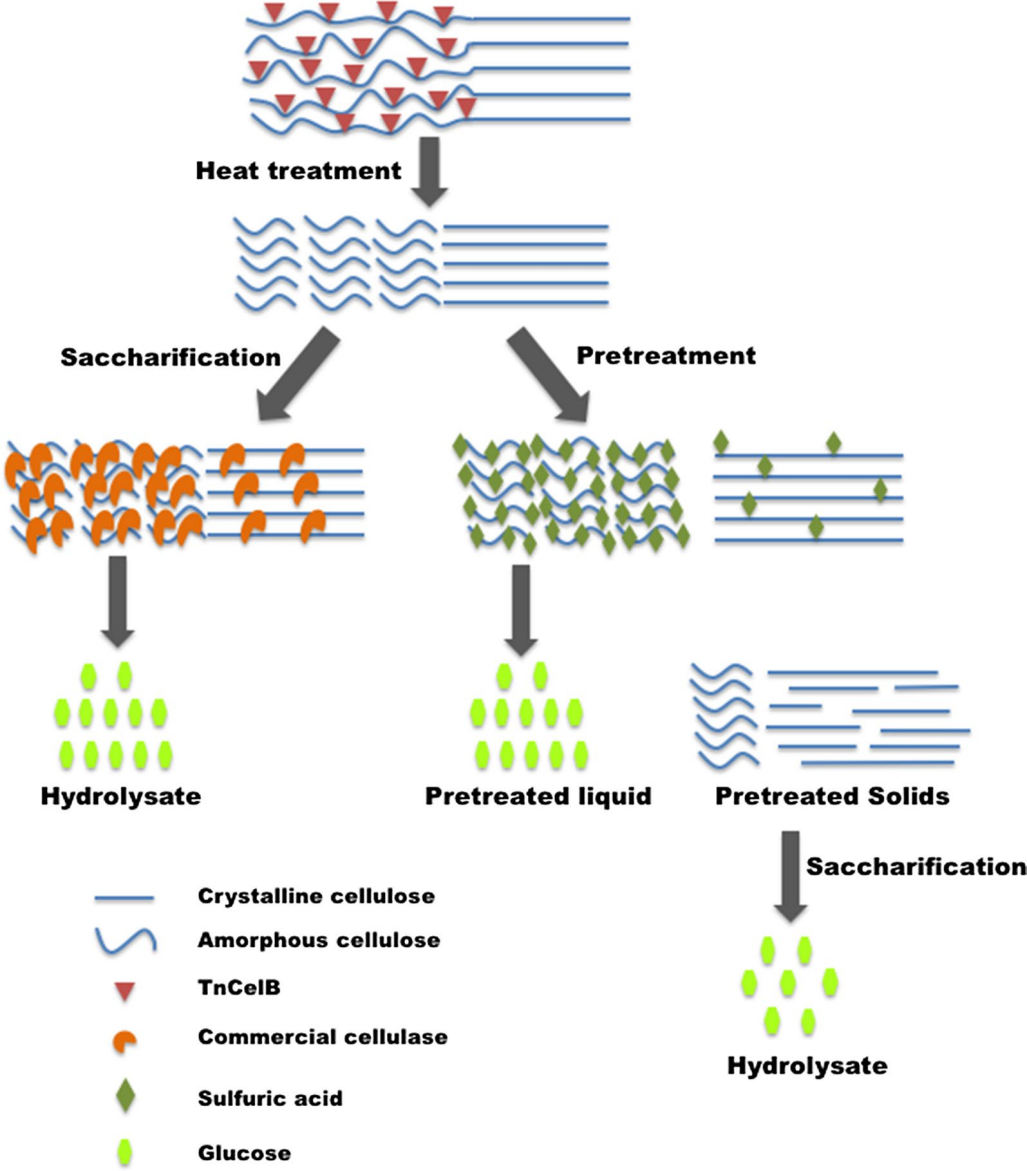
Schematic representation of predicted model of CaMV-4 event undergoing heat treatment, pretreatment, and enzymatic saccharification

When heat-treated stems underwent a mild acid pretreatment, pretreated CaMV-4 stem (solids) did not achieve higher glucan conversion rates as observed from untreated or heat-treated stem; however, CaMV-4 released more glucose into pretreated liquid during a mild pretreatment, again suggesting that CaMV-4 had a higher proportion of more easily digestible cellulose. The unique saccharification efficiencies of CaMV-4 in pretreatment (significantly lower than prior to pretreatment) could be the result of activation of high levels of *Tn*CelB during heat treatment and resultant hydrolysis of amorphous cellulose through random cleavage of β-1, 4,-glycosidic bonds resulting in abundant, easily hydrolyzed shorter glucan chains with free ends (Fig. 5). Increased glucan conversion rates of heat-treated CaMV-4 stem could be due to both of a more digestible cell wall structure and more efficient hydrolysis of shorter glucan chains by commercial cellulases (Fig. 5). Meanwhile, shorter glucan chains in heat-treated CaMV-4 are more rapidly hydrolyzed by sulfuric acid in pretreatment resulting in more glucose released into pretreatment liquid (Fig. 5). Because hydrolysis by *Tn*CelB and sulfuric acid occurs more rapidly in the accessible amorphous regions, a larger portion of crystalline cellulose is left in pretreated solids of CaMV-4 stem for saccharification and causes similar results to WT (Fig. 5).

## Conclusions

Poplar lines with moderate-to-high *Tn*CelB expression levels could serve as cellulase production platforms, as they are able to successfully produce hyperthermophilic endoglucanase. Reducing the intensity of the required pretreatment or removing the pretreatment requirement altogether would provide significant gains for affordable cellulosic ethanol production. Besides serving as enzyme providers, the poplar event with the highest *Tn*CelB expression level circumvented such pretreatment and achieved similar sugar release to pretreated non-transgenic controls, and as such, could benefit cellulose hydrolysis during ethanol production. Moreover, transgenic lines with more moderate *Tn*CelB expression levels have enhanced saccharification efficiency after *in planta Tn*CelB being activated. These characteristics could allow for the poplar biomass to be used both as a biofactory for thermophilic enzyme production as well as for the lignocellulosic feedstock itself, thus reducing required inputs. Utilizing lignocellulosic biomass to produce hyperthermophilic cellulases could benefit cellulosic ethanol production starting from biomass yields through to monosaccharide release.

## Additional file

**Additional file 1: Fig. S1.** Synthesized *TnCelB* gene sequence and splicing confirmation. **Fig. S2.** Mutated PR1a targeted GFP to apoplast. **Fig. S3.** Abnormal phenotypes of CaMV-4. **Fig. S4.** Expression level of genes involving in synthesis of monolignol (CAD), cellulose (CesA4, CesA7, CesA8, Kor), and hemicellulose (GT47C). **Table S1.** Primers used in the study.
