# Supplementary material for: Expression of a hyperthermophilic endoglucanase in hybrid poplar modifies the plant cell wall and enhances digestibility
Source: Biotechnol Biofuels. 2018 Aug 16;11:225. doi: 10.1186/s13068-018-1224-7 (PMC6094567; doi:10.1186/s13068-018-1224-7)
Supplement: Supplementary file 1 — Additional file 1: Fig. S1. Synthesized TnCelB gene sequence and splicing confirmation. Fig. S2. Mutated PR1a targeted GFP to apoplast. Fig. S3. Abnormal phenotypes of CaMV-4. Fig. S4. Expression level of genes involving in synthesis of monolignol (CAD), cellulose (CesA4, CesA7, CesA8, Kor), and hemicellulose (GT47C). Table S1. Primers used in the study. [file 13068_2018_1224_MOESM1_ESM.docx]

# Expression of a hyperthermophilic endoglucanase in hybrid poplar modifies the plant cell wall and enhances digestibility

Yao Xiao; Xuejun He; Yemaiza Ojeda-Lassalle; Charleson Poovaiah^1^; Heather D. Coleman*

Biology Department, Syracuse University, Syracuse, NY, USA 13244

^1^Current affiliation: Scion, Te Papa Tipu Innovation Park, 49 Sala Street, Rotorua 3010, New Zealand

*Corresponding author: Heather D. Coleman (hcoleman@syr.edu)

ADDITIONAL FILE 1


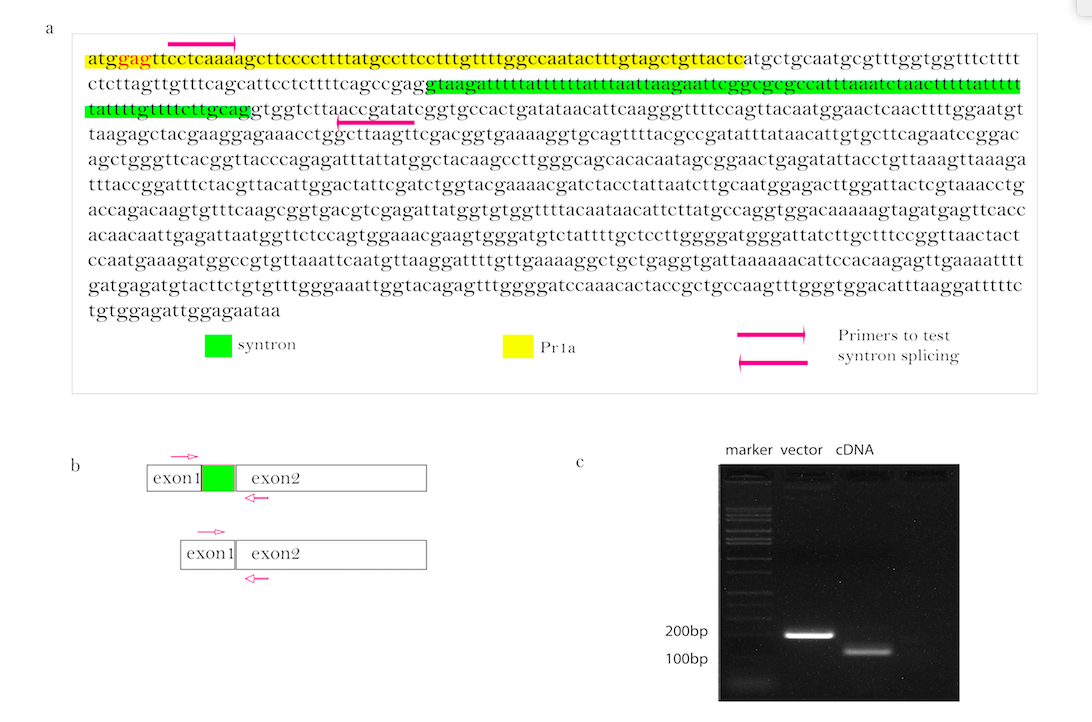


**Fig S1. Fig S1.** Synthesized *TnCelB* gene sequence and splicing confirmation. (a) Codon optimized sequence of TnCelB (b) Primer location to detect splicing of intron (c) PCR results detecting correct intron splicing


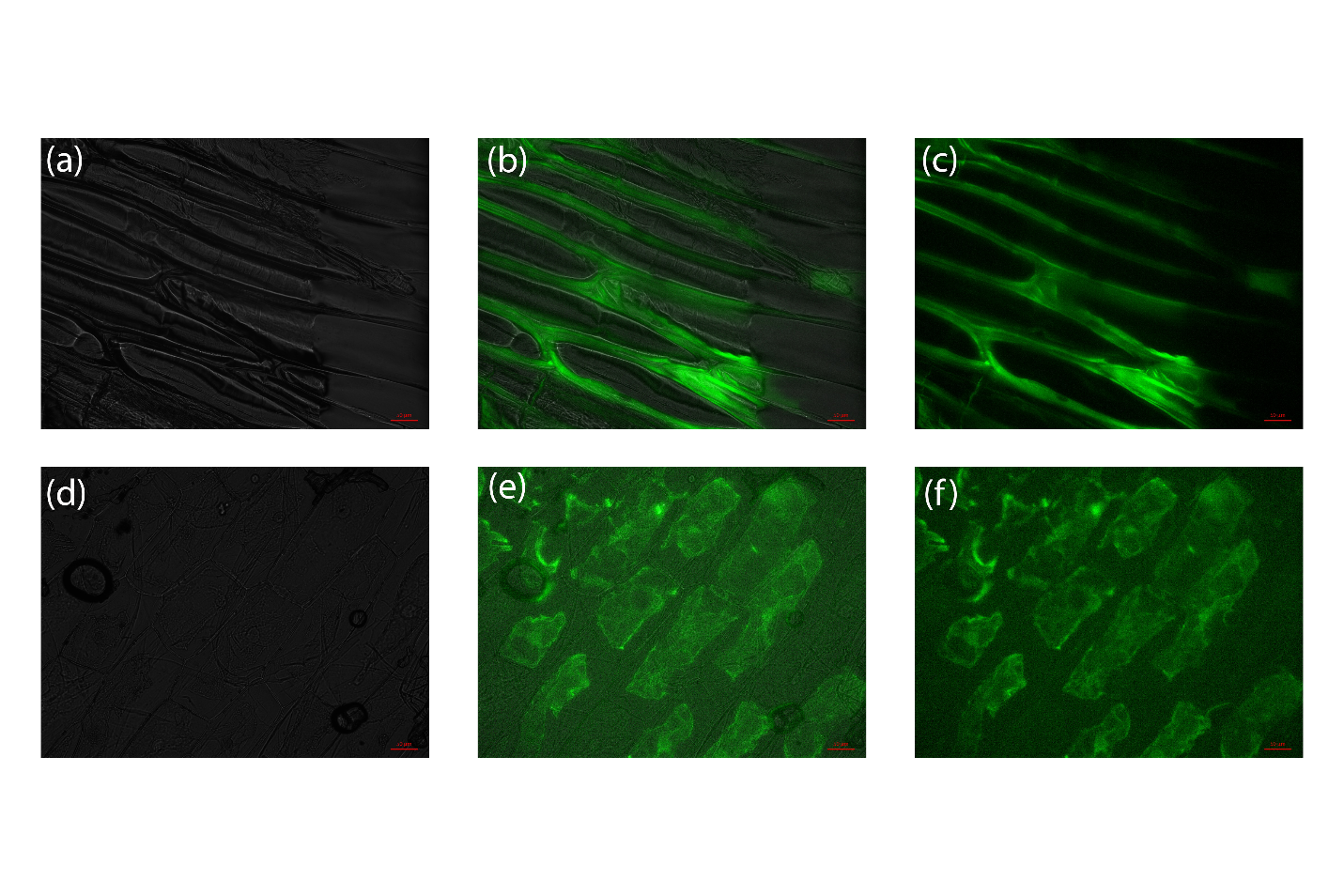


**Fig S2.** Mutated PR1a targeted GFP to the apoplast. Onion epidermal cells infiltrated with GFP fused with the mutated PR1a sequence (a,b,c) or with GFP without the targeting sequence (d,e,f); pictures were taken under white field (a,d); fluorescent light (e,f); merged (b,e)


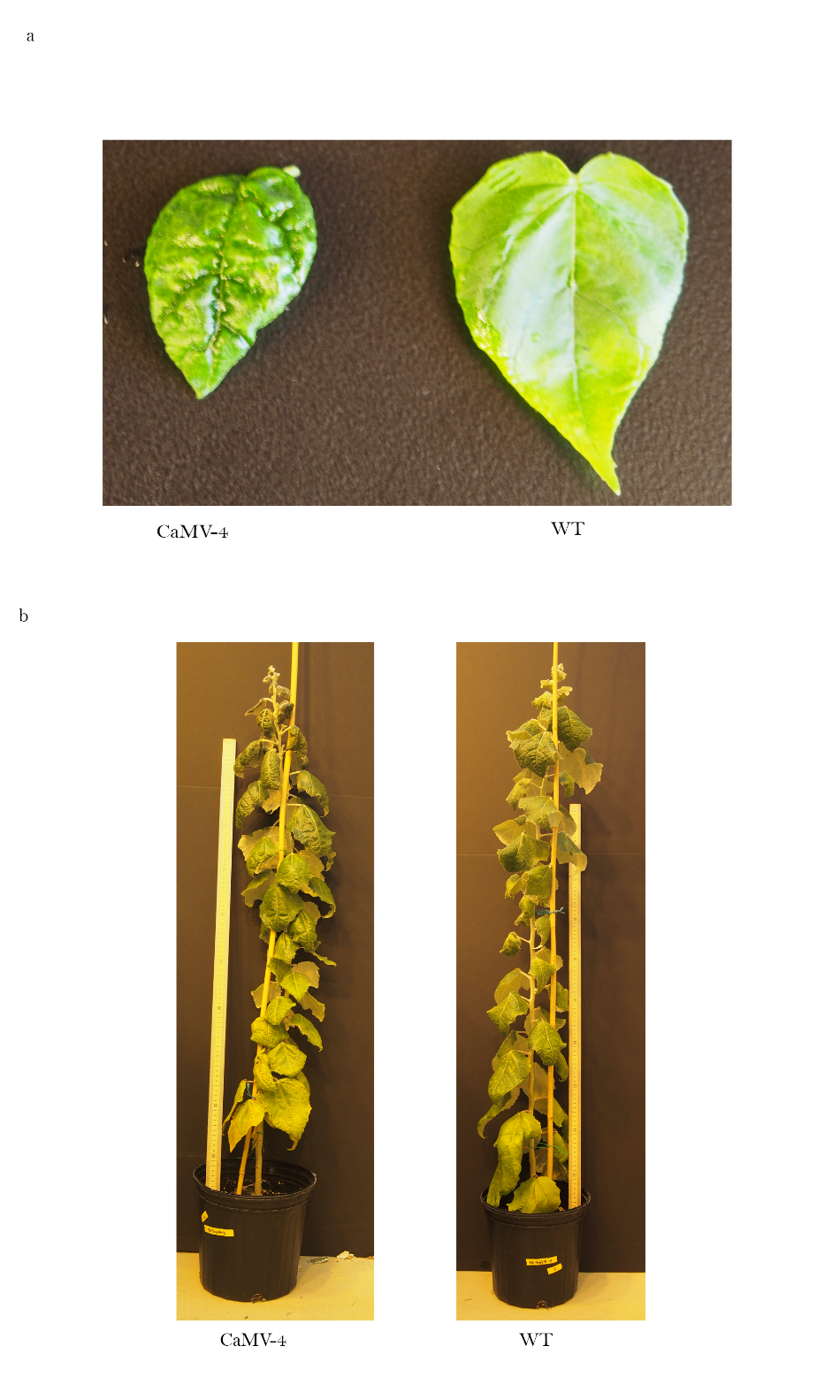


**Fig S3.** Abnormal phenotypes of CaMV-4. (a) Wrinkled leaf surface of CaMV-4 line expressing *Tn*CelB compared with WT; (b) Dehydrated apical region of CaMV-4 line expressing *Tn*CelB compared to WT

**
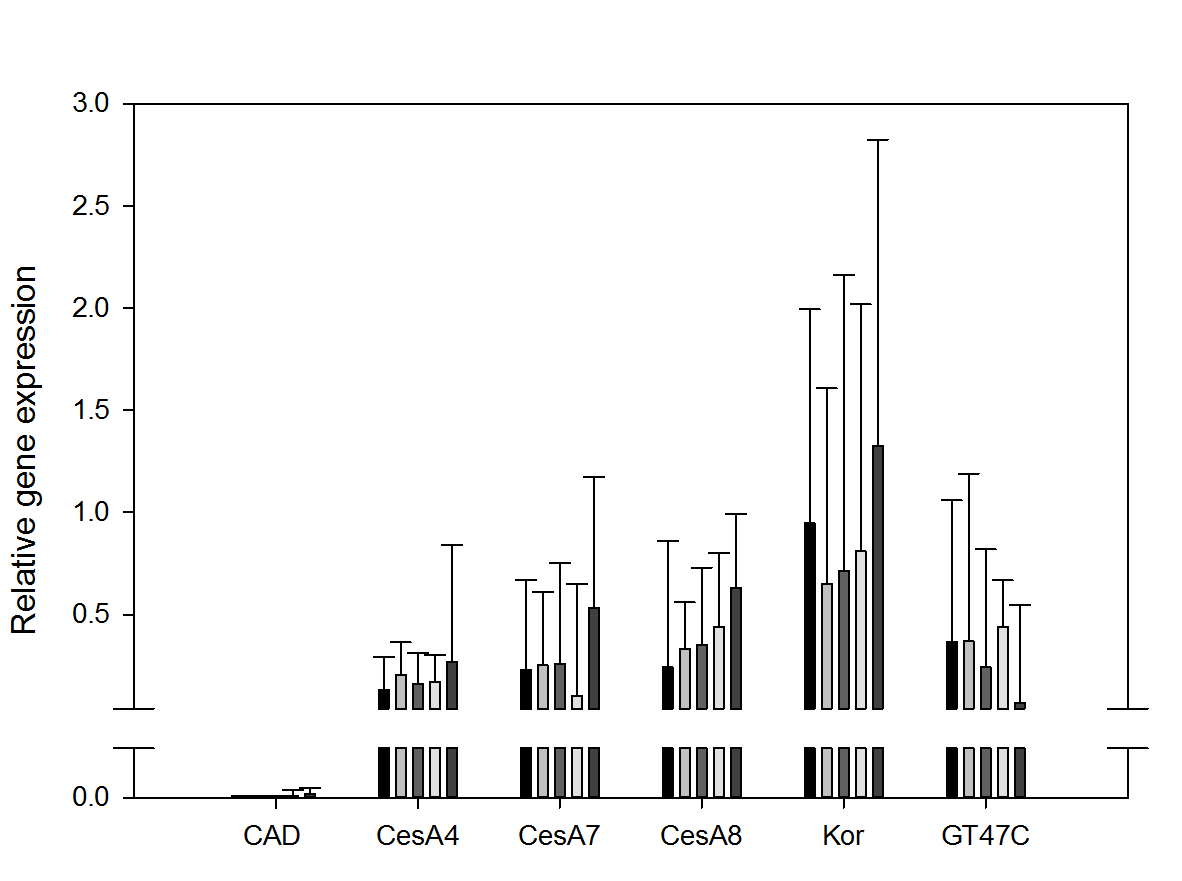
**

**Fig S4.** Expression level of genes involved in synthesis of monolignol (CAD), cellulose (CesA4, CesA7, CesA8, Kor), and hemicellulose (GT47C). Three individual plants per event were analyzed. Error bars are standard error.

**Table S1.** Primers used in the study

| **Primer name** | **Sequence** | **Note** | **Tm(°C)** |
| --- | --- | --- | --- |
| **PRSE-Fwd** | GCGGCCGCCATGGAGTTCCTCAAAAGCTTCC | Forward primer to amplify Pr1a sequence with Asn mutated to Glu, NotI site added | 65 |
| **TnCelB-Rvs-Not1** | GCGGCCGCTTATTCTCCAATCTCCACAGAA | Reverse primer for cloning TnCelB, NotI site added |  |
| **UBC11-Fwd** | GTTGATTTTTGCTGGGAAGC | Homologous locus in *Arabidopsis*: NM_001125464 | 60 |
| **UBC11-Rvs** | GATCTTGGCCTTCACGTTGT |  |  |
| **EF1β-Fwd** | GGCATTAAGTTTTGTCGGTCTG | elongation factor 1-beta 2 (LOC7489318) | 60 |
| **EF1β-Rvs** | GCGGTTCATCATTTCATCTGG |  |  |
| **TnCelB-Fwd** | ATGCGTTTGGTGGTTTCTTTTC | Forward primer for qPCR | 60 |
| **TnCelB-Rvs** | AAAGTTGAGTTCCATTGTAACT | Reverse primer for qPCR |  |
| **CesA4-Fwd** | CACAGGTTATCCCACTTTTGCT | JCI accession number: eugene3.00002636 | 60 |
| **CesA4-Rvs** | CATACGCTTGCTTGCTAACAGA |  |  |
| **CesA7-Fwd** | CAAGCAATGTGGACTCAACTGTTA | JCI accession number: gw1.XVIII.3152.1 | 60 |
| **CesA7-Rvs** | AAGCAGGATGCACATGTATCTTCT |  |  |
| **CesA8-Fwd** | AAGCACATATCGCTGTCAGTATTTA | JCI accession number: eugene3.00040363 | 60 |
| **CesA8-Rvs** | TTCAACACAATCAAAACCTGTATTT |  |  |
| **Kor-Fwd** | GCAGCAAAATCATCTTACCAA | GenBank: AY535003.1 | 60 |
| **Kor-Rvs** | GGATTGACAAGAACACCATAT |  |  |
| **CAD1-Fwd** | GCAAGCTTATTCACTGAACAACAAT | JCI accession number: estExt_Genewise1_v1.C_LG_IX2359 | 60 |
| **CAD1-Rvs** | AAAGCAAAGACACACTGTCACATTT |  |  |
| **GT47C-Fwd** | TCCTCCAACTCCACTTTCATTC | Accession numbers: PoGT47C (DQ899955) | 60 |
| **GT47C-Rvs** | AGCCAGTCCGTGTTGTATTT |  |  |
| **CaMV-Fwd** | CTGCAGGGCTAGAGCAGCTTGCCAAC | Primer to check presence of expression cassette | 55 |
| **TnCelB-Rvs-Not1** | GCGGCCGCTTATTCTCCAATCTCCACAGAA | Reverse primer for checking presence of expression cassette |  |
